# Oral health-related cultural beliefs for four racial/ethnic groups: Assessment of the literature

**DOI:** 10.1186/1472-6831-8-26

**Published:** 2008-09-15

**Authors:** Yogita Butani, Jane A Weintraub, Judith C Barker

**Affiliations:** 1 Center to Address Disparities in Children's Oral Health at the University of California, San Francisco, CA, USA

## Abstract

**Background:**

The purpose of this study was to assess information available in the dental literature on oral health-related cultural beliefs. In the US, as elsewhere, many racial/ethnic minority groups shoulder a disproportionate burden of oral disease. Cultural beliefs, values and practices are often implicated as causes of oral health disparities, yet little is known about the breadth or adequacy of literature about cultural issues that could support these assertions. Hence, this rigorous assessment was conducted of work published in English on cultural beliefs and values in relation to oral health status and dental practice. Four racial/ethnic groups in the US (African-American, Chinese, Filipino and Hispanic/Latino) were chosen as exemplar populations.

**Methods:**

The dental literature published in English for the period 1980–2006 noted in the electronic database PUBMED was searched, using keywords and MeSH headings in different combinations for each racial/ethnic group to identify eligible articles. To be eligible the title and abstract when available had to describe the oral health-related cultural knowledge or orientation of the populations studied.

**Results:**

Overall, the majority of the literature on racial/ethnic groups was epidemiologic in nature, mainly demonstrating disparities in oral health rather than the oral beliefs or practices of these groups. A total of 60 relevant articles were found: 16 for African-American, 30 for Chinese, 2 for Filipino and 12 for Hispanic/Latino populations. Data on beliefs and practices from these studies has been abstracted, compiled and assessed. Few research-based studies were located. Articles lacked adequate identification of groups studied, used limited methods and had poor conceptual base.

**Conclusion:**

The scant information available from the published dental and medical literature provides at best a rudimentary framework of oral health related ideas and beliefs for specific populations.

## Background

Culture is often defined as coherent, shared patterns of actions or beliefs specific to named groups of people that provide basic life roadmaps or social contexts, defining behavioral norms and interpersonal relationships as well as unwritten rules for proper living. A key element of social context is the patterned process of people making sense of their world, and the (conscious and unconscious) assumptions, expectations, and practices they call upon to do so [1]. Culture organizes the group's norms of family life, birth, childrearing, aging, and death [2] as well as their recognition of illness and care-seeking practices around health or medical conditions. Sometimes these beliefs and practices can facilitate or act as barriers to accessing health care services.

Reports on the state of oral health of the United States populations suggest that people from specific ethnic minorities often have poor oral health status [3-5]. Being a part of an ethnic minority group does not inevitably lead a person to have poor oral health. It does suggest, however, that there may be certain cultural beliefs and practices common to the people in these groups which influence their oral health status, such as values placed on having healthy primary teeth or expectations about preventive or therapeutic interventions. Cultural factors may have important implications for an individual's own health and those of others for whom they provide care, such as children and the elderly [6-9].

Race/ethnicity is a marker for oral health status. Underlying cultural beliefs and practices influence the condition of the teeth and mouth, through diet, care-seeking behaviors, or use of home remedies, for example. It is important to note that among, and within, all racial/ethnic groups there are substantial differences in beliefs and behaviors, which can lead to varying health status. Such differences are often associated with demographic characteristics. For example, Latinos from Puerto Rico share certain characteristics with Latinos from El Salvador yet may have very different health status, attitudes towards and patterns of use of health services [10,11]. Similarly, Mexican and Mexican-American populations have different health experiences and patterns of service use, due in part to socio-cultural differences. Research on poor health outcomes generally examines deterrents such as high cost, lack of insurance and availability of services, but often aspects of cultural ideas and practices are also suggested as additional deterrents [12].

Worldwide, there are multitudinous distinct ethnic minority and cultural groups [13]. Some groups are indigenous to the country in which they are found; others are migrants. In the US, for example, there are over 500 federally recognized indigenous American Indian tribes and bands. There are multiple other ethnic communities too along with immigrants from every region of the world. In recent decades, for example, people from North Africa, Turkey, the Middle East, and Asia have moved not just to US but also to France, Germany, Scandinavia, Britain, South America and Australia, and other countries. Every ethnic group has its own set of beliefs and attitudes towards oral health care.

### Focal ethnic groups

To explore the vast topic of cultural beliefs and practices in relation to oral health, this literature assessment focuses attention on four population groups that reside in the US: African American, Chinese, Filipinos and the Hispanic/Latino populations. The selection of these groups was based on their demographic importance, geographic dispersion, family composition and the proportion of the population experiencing socio-economic hardship [14], all factors that may be associated with populations most likely to be experiencing oral health disparities compared to the mainstream US population [5]. Nationally, African-Americans, numbering 37 million in 2001, and Hispanic/Latinos, reaching 44 million, were the largest minority groups [15]. In 2000, Chinese and Filipino were the most frequently reported Asian ancestries in the U.S. [15]. This review was conducted in conjunction with a qualitative study undertaken in California [7] of cultural factors associated with oral health. Within California, the most populous state, Latino and Asian groups are increasing fastest, with Hispanics comprising almost a third, Chinese comprising 2.9% and Filipinos 2.7% of the nearly 34 million total population in that state in 2000 [14]. All four groups in the US as a whole and in California are demographically young: at least two-thirds of these populations is aged 18 years or less, and except among African-Americans, at least two-thirds of the population in each group speaks a language other than English when at home [16].

All available, retrievable literature on culturally specific health beliefs around oral health was gathered for these four population groups to produce an assessment of the adequacy of the published literature in representing that population's oral health culture. This article examines and evaluates the state of knowledge relevant to the broad topic of how culture affects each group's oral health. The primary research question was: *Does the published oral health literature adequately document cultural beliefs and practices for each specific group? *A series of sub-questions further framed this assessment: a) How much literature is there? b) What types of literature exist, and what methodological approaches and research designs have been used in examining cultural issues in relation to oral health? c) What content or information has been presented? d) How useful is present knowledge for guiding clinical understanding?

## Methods

The literature was scrutinized in relation to five major domains of cultural information, four of them specific to oral health: 1) basic conceptual models or ideas about health and disease, 2) help-seeking for oral conditions, especially use of folk or traditional health remedies, 3) diet, 4) beliefs and practices about teeth and the oral cavity, and 5) oral hygiene practices. Collectively, these domains shape people's cultural beliefs and practices related to oral health and reasons for seeking dental services for themselves and their dependents, such as the elderly and children. Later, we use these domains to organize and present the topical findings.

### Literature search

To identify literature on cultural beliefs and practices regarding oral health, the electronic database for the National Library of Medicine (PUBMED) was searched for all relevant literature published in the English language spanning a twenty-seven year period, 1980 to 2006. The search was limited to the four ethnic groups mentioned above. This time period was selected because a system to collect data on the ethnic background of populations in the US was not in place until the late 1970's e at [17]; hence the 1980 date to ensure that system was well established. The period ends at 2006, as this was the latest year in which complete data was available for an assessment performed in 2007.

Keywords and MeSH headings were used in many different permutations to identify relevant literature. Descriptors for each of the four ethnic groups were sequentially combined with each term in the following two lists: (1)"traditional medicine", "folk medicine", "cultural beliefs", "ethnicity", "racial", "traditional practices" AND (2) "dental", "dentistry", "oral", "teeth", "gums", "caries", "periodontal". Because using all the search words together in a serial manner yielded significantly lower numbers of relevant articles, each ethnic group's name was combined with one search word from each of the two lists in a step-wise fashion. One example is (Chinese) AND (traditional medicine) AND (dentistry). Search words used are reported in Table 1.

**Table 1:** Summary of keywords and MeSH headings used to identify relevant literature

	Keywords or MeSH terms
1	(African American/Black) (traditional medicine, folk medicine, cultural beliefs, ethnicity, racial, traditional practices), (dental, dentistry, oral, teeth, gums, caries, periodontal)
2	(Chinese/Oriental Asian) (traditional Chinese medicine, folk medicine, cultural beliefs, ethnicity, racial, traditional practices), (dental, dentistry, oral, teeth, gums, caries, periodontal)
3	(Filipino/Philippines) (traditional medicine, folk medicine, cultural beliefs, ethnicity, racial, traditional practices), (dental, dentistry, oral, teeth, gums, caries, periodontal)
4	(Hispanic/Latino) (traditional medicine, folk medicine, cultural beliefs, ethnicity, racial, traditional practices), (dental, dentistry, oral, teeth, gums, caries, periodontal)

The search words were pre-tested and refined in order to select the final search terms (by YB and JCB). One author (YB) conducted the PUBMED search using the above strategy on two separate occasions, with identical lists of publications resulting. The titles and abstracts (when available) were read and appropriate articles selected and retrieved for review of the article in its entirety. These selection criteria, developed by YB and JCB, were (a) the article must mention at least one specific belief or practice, and (b) had to name the ethnic group studied. Articles that spoke generically of "cultural influences", or stated "ethnic" or "minority groups" without specifying the name of the population, were excluded. A data abstraction form, available from the corresponding author, was developed to ensure rigorous abstraction of all relevant data. This form included headings to collate information on the name, location and socio-demographic composition (e.g., age, income level) of the ethnic groups studied, sample size, study design, and beliefs reported. Descriptive findings are reported.

### Inclusion and exclusion criteria

To capture as much relevant information as possible, all types of literature were included, such as reports of original research, reviews, commentaries, comments, letters and opinions because the volume of publications discussing cultural issues is very slim. Publications were excluded if they reported only quantitative epidemiologic data with no reference to specific beliefs or practices regarding oral health for the group(s) under study, or if the reports were generic overviews linking cultural issues to oral health, but information specific to these four ethnic groups was not presented or could not reasonably be deduced from the original report. A secondary search of the bibliography of the appropriate selected studies was also performed to identify any relevant articles that were not identified by the primary PUBMED search. The relevant literature for each population group was assessed for methodological approach, the nature of the cultural information presented, and its degree of relevance to oral health or the practice of dentistry.

For groups other than the African-American population, the search strategy was not limited to studies published or conducted in the US. For the Chinese, Filipinos, and Hispanic/Latinos immigrant populations, all the literature discovered was reviewed and, if relevant, was included, irrespective of place of study. The three groups of interest are all recent (mainly within the past 30–50 years) immigrants with strong connections still to their countries of origin, connections that can serve to strengthen resistance to changes in beliefs and attitudes. Several reasons support the decision to include relevant studies from outside the US. Changes due to acculturation to a new environment occur in staggered fashion, with change in behaviors occurring long before changes in underlying beliefs. As noted in the introduction, changes in diet, types of health care utilized, access to these services, financial stability, social status and so forth, can – and often do – all change dramatically and rapidly soon after migrating to a new country. Cultural knowledge, beliefs and attitudes about health and sickness, however, especially of causes, recognition and consequences of illness, and proper treatment, change far more slowly, often not until the next generation is born and raised in the new country of residence. So, some included studies refer to these populations in their homelands or in the United Kingdom or Singapore or elsewhere. For African-Americans, however, the literature was restricted to include only studies referring to descendents of populations originating in Africa and brought to the United States as slaves before 1860. Publications referring to other Black or African immigrant populations in the U.S. (e.g., Ethiopian, Black South African, Afro-Caribbean groups) were excluded because compared to African-Americans these groups have had very different socio-political histories and experiences, and have distinctly different cultural beliefs and practices.

This review is intended to assess the knowledge or understanding of the cultural beliefs about oral health for these four selected ethnic groups, and thereby to assess more generally knowledge of cultural issues in the oral health literature.

## Results

**Figure 1:**
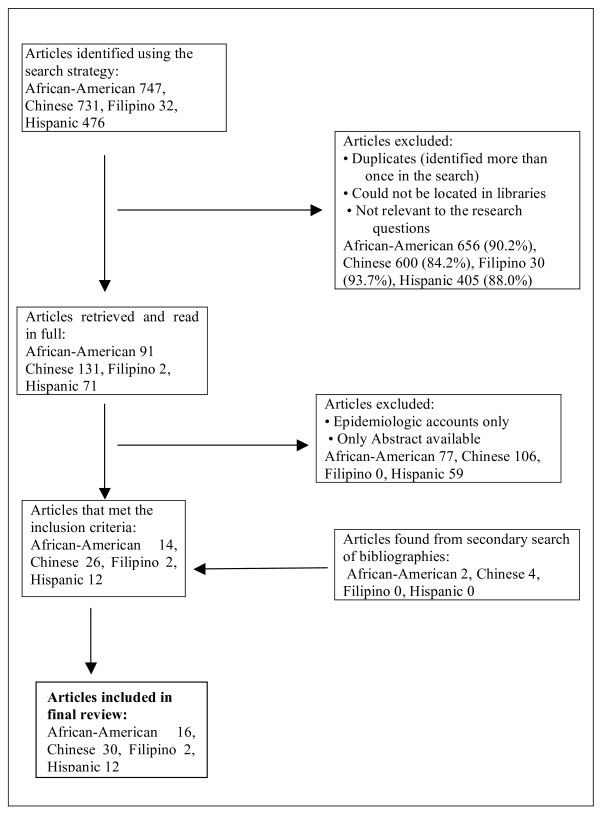
Search Strategy and Results of Literature Identification.

A schematic representation of the search strategy and the articles found is presented in Figure 1. A total of 60 studies were included in this review: 16 articles about African-Americans [18-33], 30 about Chinese [12,34-62], two about Filipinos [6,63] and 12 about Hispanic/Latinos [10,23,64-73]. Figure 1 summarizes the search strategy and the quantity of included and excluded literature. For every population group, more than 80% of the literature initially identified was excluded as it either did not discuss at all or discuss in any detail any connection between cultural ideas and oral health.

### Overall assessment of the literature reviewed

Each piece reviewed exhibited one or more deficits which detracted from its overall usefulness with respect to the primary purpose of this article. While more recent works tended to display fewer and less serious deficits, the available literature on this general topical area is not yet extensive or robust. Deficits were of several types: 1) lack of conceptual clarity and specification of the population under examination; 2) use of a limited set or inappropriate type of method; and, 3) a sketchy coverage of pertinent topics.

#### Lack of conceptual clarity and specificity

Authors frequently generalized, even stereotyped, population groups. Little account was taken of the considerable within-group diversity that exists in all populations. This diversity is due to socio-economic class, income, education, geographic or regional location, religion, language, and history of migration (to US), or level of acculturation. There is also heterogeneity in specific beliefs, values and practices around health, prevention of disease, help-seeking and self-care practices. Rarely do cultural beliefs come in just one standard form but usually in a variety of closely related forms; compared to men in the group, women, for example, may hold similar but nonetheless variant or more elaborated views on health-related issues, especially with respect to children, because women are often deemed to be primarily responsible for monitoring and ensuring a family's health and well-being. Other socio-demographic factors, such as age, can have a similar effect in producing with-in group diversity in ideas and practices. Further, published works usually lack detailed demographic information about the study population; and make very poorly explicated connections to other aspects of culture. Many works proved to be old, anecdotal, and offer little evidence of how cultural beliefs might have metamorphosed in the years since the study was conducted. Often studies are overly generic, with little acknowledgement or identification of crucial features that make for important cultural distinctions. Language is one such feature; for example, which of many dialects did a Chinese population under study actually speak? – Cantonese, Mandarin, Toishanese, Fukienese, and so forth. The name of the specific minority or ethnic group studied is rarely provided; for example, did the Hispanic/Latino group being researched originate in Cuba, Puerto Rico, the Dominican Republic, Mexico, or Nicaragua? What religion does the study group espouse; so, for instance, does the Filipino group comprise mainly Protestant, Catholic or Muslim adherents and so come from different geographic regions of their homeland, speak different languages, have different beliefs and practices, and different access to dental services, education, economic resources? How does the migration experience of poor Salvadoran or Guatemalan refugees, escaping war and torture, differ from that of Mexican farm workers or Puerto Rican or Cuban migrants? Are the migration experiences of poor Hispanics comparable in any way to those of wealthy Chinese from Hong Kong? Where geographically does the group live? For example, familial experiences and present-day connections to the US health care system are different for African-Americans from rural Louisiana compared to those in middle-class urban areas or in inner-city slums.

#### Limited methods

A rather limited – and not always appropriate – repertoire of investigative methods has been brought to bear on the issues of concern in this review. Some studies resorted to use of structured questionnaires and telephone surveys, not the best means to explore cultural phenomena that are not already fairly well understood. While many works did use qualitative methods, very little ethnographic work was represented in this literature and there was a tendency to prefer a focus group method over other styles of qualitative inquiry, such as face-to-face, individual interviews. Too often the method used was inappropriate to the kinds of questions posed or the general intent of investigations. Focus groups, for example, are frequently employed but this approach is best when used to establish consensus of opinion within a group and not for eliciting variations in beliefs and experiences. Relatively few authors appeared to have extensive familiarity with the logic or procedures of qualitative inquiry in general.

Method of inquiry is intimately connected to underpinning theory. However, the social or psycho-social theory guiding the study procedures and data analysis were usually absent. Disjuncture between the epistemological assumptions and processes of a study's topical intent/procedures and the implicit socio-psychological theory/method employed, is a common although still vastly under-recognized problem in the kind of biomedical investigations reviewed here [33,60].

#### Topical content

Some works were initially identified as possibly relevant but ultimately excluded from this review, usually because they made unsubstantiated comments or assertions in the text. Thus, their inclusion was not justified. Epidemiological accounts were not included if they posited "culture" as an explanation for findings but the specific aspects of culture were not further revealed.

There is little discussion in the literature on basic health care beliefs, ideas about health and disease, health care seeking behaviors, diet, and cultural beliefs about the function of teeth at various life stages. Overall, very few studies were found that discussed cultural beliefs related to oral pain and methods for relieving it. It may be, however, that pain and swelling in any region of the body is treated similarly and therefore that there is no distinct reference to folk or other remedies for treating oral conditions. Some recent studies have found that caregivers of young children frequently feel first teeth are not as important as permanent teeth [7,60,74]. Tooth loss in old age appeared to be commonly accepted as a norm in all four cultural groups examined [18,20,35,36,55,63]. The timing of care seeking (whether for preventive, routine or emergency care) has rarely been studied in association with cultural beliefs. Cultural beliefs related to preventive strategies were rarely discussed; indeed, some of these cultural groups did not have a strong preventive orientation. More detailed understanding of culture as it relates to oral health is needed because minority groups have high oral disease rates, and are dentally underserved.

Since the information reported in the literature is so slim, and cultural beliefs and practices diverse even among these ethnic/racial groups (e.g., by socio-economic class, religion or language), generalizing the results presented below should be done with extreme caution. When the data in the original literature report were not rigorously collected or findings were not replicated in other studies, the actual content of beliefs or practices has not been reiterated here. Overall, in the literature reviewed, the most comprehensive, detailed accounts of culturally-based ideas and practices of direct relevance to oral health come from studies of Chinese populations. Some relevant works were located for African-American and Hispanic/Latino populations; almost no work exists on Filipinos. Sparse, poorly connected and incomplete as they are, the major findings for each group are summarized below, organized roughly by the five domains identified as pertinent in the introductory section of this article.

### African-American

Most African-Americans subscribe to broadly the same set of ideas as the dominant white/Anglo-European population in the US with some continuing ideas from previous eras, when self-care and "folk" practices were the only available resorts. Despite comprising a large minority group, relatively little work has investigated African-American (oral) health beliefs and practices. Additionally, most reports in the literature on African-Americans examine cultural beliefs and behaviors among the low-income sector of the population and not those with higher incomes.

No discussion was found of African-American cultural beliefs about the function of teeth at various ages, from infancy to old age; associations between physical and oral health; or about the relationships between diet and oral health in late childhood or adulthood. One study from the Midwestern U.S. claims that the mother's diet during pregnancy is thought to be an essential factor in the later causation of "soft teeth" or dental caries in the child [18]. Some works suggest that African-Americans believe that caries can progress to become a serious problem if it is not treated [21,22]. Most (70%) African-American respondents in one study believed that pain in the oral cavity was an early symptom of oral cancer [25]. Among African-American elders, oral pain was often associated with needing dental care [30].

Norman and colleagues report that parents who had fatalistic beliefs (such as: most children eventually develop dental cavities) have less knowledge about their children's oral health needs. They also are less likely to brush their child's teeth and seek dental care [31]. Broder and colleagues report the frequent use of bottle with juice, soda or other sweetened drinks by children at bed-time [32]. Kelly and colleagues compared African-American parents who utilize or do not utilize dental services for their children, reporting contrasting beliefs between the two groups. African-American families that visit a dentist regularly tend to have stronger preventive beliefs, were more knowledgeable about infant gum and tooth care and long-term consequences of oral diseases. Some of the parents not utilizing dental services expressed dissatisfaction with the care they had previously received, an attitude that shaped their present practices [33].

Commonly reported home remedies comprised methods to relieve pain and swelling (e.g., use of cotton balls soaked in aspirin solution, alcohol or salt water) or to relieve toothache pain (e.g., cotton balls soaked in turpentine and sugar or oil of cloves) and self-medication with over-the-counter pain medication [18,19,33]. Sketchy reference was also made to cleaning teeth in order to maintain good oral hygiene [18,20-23], although flossing has also been reported [22,24]. Practices such as self-medication could vary by geographic location and be different among younger or more middle class African-American adults due to their greater education or income levels and willingness to access professional dental care [18]. Traditional practices, however, could continue especially among low-income African-American families and those without dental or health insurance.

Dental visits are said to be mostly problem – rather than prevention-oriented [26,30,33], with women more likely than men to see a dentist on a regular basis [27]. Consistent with a strong cultural focus on spiritual practices and religious participation, African-American church-attendance was related to concerns about oral health status and utilization of dental services [28,29]. Treatment preferences are said to be for extraction rather than to save a tooth, although this claim is likely to be strongly affected by income and socio-economic status [19,24].

Gilbert and colleagues report self-extraction is a commonly employed method of relieving tooth pain among African-Americans in Florida [24]. Esthetic appearance or "looking good" is commonly associated with having good teeth [18,26], and is said to motivate some African-Americans in Detroit to seek professional dental treatment [22].

### Chinese

Underlying philosophies and conceptual frameworks ground Chinese health beliefs. Traditional Chinese Medicine (TCM) emphasizes the universe-human body relationship [34,75]. Chinese beliefs about health and illness management are holistic, woven into the social and cultural fabric of daily life, conceptualized within the context of yin-yang, hot-cold, and dry-wet balances, as well as qi and holism [37-39,75]. Another theory the Chinese use is the Meridian Theory, which assumes that any disorder within a meridian or energy pathway generates disharmony along that meridian. For example; maxillary toothache may result from a disorder of the stomach meridian; likewise mandibular toothache may result from a disorder of the large intestine meridian because the large intestine and the mandible run along the same energy channel [40]. Inadequate sleep or stress affects the meridian involving the stomach and are also believed to cause gum disease [35,42].

Based on the concepts of TCM, the Chinese believe that tooth health depends on the condition of the kidneys. The kidneys determine the condition of the bone, as the bone is filled and nourished by marrow, which is believed to derive from the vital essence of the kidneys. The teeth are considered the odds and ends of the bone. Therefore, problems such as loosening of teeth are considered to be an expression of the imbalance between the two vital forces (yin/yang) in the kidneys. Similarly, the gums are related to the stomach via meridians through which vital forces (yin/yang) move. Gum inflammation is believed to result from intense heat or flaring fire in the stomach [37,43].

The Chinese tend to use traditional medicine in conjunction with western medicine for minor, well-understood or common health problems; for uncommon or more serious ailments they often seek biomedical treatment [35,37,59]. Western medicine is considered good for the treatment of symptoms while Chinese medicine is believed to be more effective in curing the disease [37]. TCM is considered culturally appropriate, holistic, convenient, cost effective, and without side effects. It can be used by people who fear going to the dentist [59]. TCM is also commonly used in response to oral mucosal lesions and periodontal disease [59]. These ideas lead to a strong reliance on self-care, which leads to delay in seeking care according to biomedical dental standards [38].

From a clinician's point of view, treatment based on TCM is initiated based on etiology. Periodontal diseases may be treated in different ways based the presentation of the periodontal tissue. For example, if there is inflammation and bleeding of gums, suppuration and halitosis, this combination is believed to be due to 'heat in the stomach'. However, if there is tooth mobility, a diastema due to tooth migration, exposure of root surface due to gingival recession, sensitivity and slight redness, this combination is believed to be due to 'deficiency of the kidneys' [34]. The presentation of the tongue is considered an important diagnostic tool in TCM [44]. For example, pale or normal tongue with wet sides accompanied by loss of appetite, distension of the abdomen, soft stools, general fatigue or malaise, and impaired memory indicates 'spleen chi deficiency'. A tongue with red sides accompanied by anger, insomnia, irritability, aching pain in the head and neck and stress is considered to be indicative of 'liver fire rises' [44].

Drinking a cooling tea or taking herbal medicine is a common practice in treating 'hot' gum diseases [35,37,43,44]. Foods or medicines are described as 'hot' or 'cool' not on their physical temperature so much as on their effect on the disease state and symbolic associations with other resources. Powdered alum, musk and frankincense, for example, are regarded as 'cool' materials and so used to treat 'hot' gum disease [34]. Now classed as quackery, a prevalent idea and connected practice in ancient China and in parts of rural China until recently [46], was that of caries being due to a burrowing worm and the use of leek seeds soaked in sesame oil to drive the tooth worm out of carious lesions.

Wong and co-workers in the US [60] noted that although parents would frequently use a traditional remedy for themselves, they were less likely to use it for their child. Most parents brought the child to the dentist or pediatrician in response to pain [60]. This research team reports a widespread belief that treatment for primary teeth was not essential, and that many more conservative-minded Chinese consider Western medicine aggressive and in some instances used too extensively [60]. Young Chinese adults and teenagers residing in the UK report trusting dentists, who are thought capable of relieving most dental problems; elderly Chinese, however, do not trust the dentist's competence in the same way [54,55,58].

Reported in the literature for Chinese populations are a variety of widespread preventive dental practices. These include: mouth rinsing and use of toothpicks after meals [12,40,41,44,47,48], use of salt to swab the teeth or in solutions for gargling to prevent gum disease [34] and scraping the tongue in the morning [47]. Other oral health beliefs reported in the literature are: tooth problems being considered a sign of aging, a natural process that may not be reversed or altered [35,47]; frequent childbirth is associated with tooth loss due to calcium deficiency; giving birth to a child with teeth is a sign of bad luck; and parents who have teeth in advanced old age is considered to bring bad luck to their children [12,40,47]. Piercing the tongue and cheeks to please the Gods is a religious practice for some Chinese people [49].

These traditional practices and beliefs should not be generalized to all sectors of the Chinese population, however; they may not be practiced in all regions of China, nor among all language groups, social classes, and so forth. Urban dwelling Chinese adolescents residing in China tend to have beliefs shaped by modern scientific knowledge mixed with traditional Chinese theories of disease and health [62]. For example; gum disease was believed to have been caused by a combination of the following: mixing hot and cold foods, incorrect tooth cleaning, unhealthy diets and general illness [62].

A concern for esthetics, appearance and freedom from pain often motivate Chinese people to maintain good oral hygiene [12,36,47,50]. Pain or troubles with teeth are common reasons for seeking oral health care [36,48,50,54,55,60]. White wine or vinegar applied directly to the hurting tooth is believed to ease tooth pain temporarily [60]. Acupuncture is occasionally used for local dental anesthesia in China [51]. There is widespread belief that tooth brushing is necessary to prevent caries [39,57,58]. The Chinese consider the appearance of teeth psychosocially important, and able to influence social interaction. A person with carious or discolored anterior teeth, for example, is considered to have low intellectual competence [51]. Orthodontic treatment to enhance appearance is common among Chinese residing in the US [52,53].

### Filipino

Very little published information about oral health beliefs or practices is available for Filipinos. An early report [63] from the Philippines noted that information and values regarding general health as well as oral health are usually passed on from the elders in the family. A more recent work reports that among more modern, affluent or urban-dwelling Filipinos this source of information is supplemented, even supplanted occasionally, by knowledge learned in school, and from reading and classes related to child rearing and Lamaze [6]. Migrant Filipinos residing in Saipan, Micronesia indicate that parents' (mostly the mother) fear dental treatment without anesthesia, and personal negative experiences in the dental office prevented them from seeking care for their children [6]. Among the low-income Filipino population especially, however, cost rather than fear was the most common reason for not seeking professional dental care [6].

### Hispanic/Latino

Despite being the largest minority group in the US, there has been relatively little research that documents Hispanic/Latino beliefs and practices in relation to oral health. Hispanic/Latino cultural beliefs regarding underlying basic concepts of health, help-seeking behaviors, function of teeth at various ages, from infancy to old age, and associations between physical and oral health are sparsely reported in the oral health literature. The literature suggests that Mexican Americans residing in various parts of the US lack adequate knowledge about the role of fluoride in caries prevention, and lack knowledge about the connection between oral health and consumption of sweets and frequent snacking [64-67]. Some reference has been made to a belief among Mexican immigrants in rural California that diarrhea and or fever is common when the child's teeth erupt [10,70]. Certain dietary and infant feeding practices are commonly reported by Mexican Americans and Puerto Ricans, such as putting an infant to bed with a bottle of sweetened liquid, giving a child a pacifier dipped in honey, or sharing eating utensils among siblings and caregivers [22,64,66,68,73]. There is reference to Mexican American migrant farm workers in Washington state that caregivers prefer to do something extra (like using fluoride varnish) for the child's oral health rather than to alter the child's feeding patterns that may cause sleep and familial disruptions [69]. Puerto Rican parents may be more likely to follow advice from elders in the family regarding a child's feeding, advice which at times may conflict with the medical advice provided by pediatricians [73]. There are reports that elderly Latinos residing in Wisconsin believe the purpose of tooth brushing is to freshen breath rather than to prevent oral disease [71]. Hispanics/Latinos from Wichita, Kansas fear loosening teeth due to oral prophylaxis [72] and Latinos from the Washington DC area believe that tooth loss is inevitable as one gets older [67]. Puerto Rican parents are said to value the aesthetic appearance of white, healthy looking teeth and so encourage their children to maintain "a bright smile" [73] but reportedly do not seek regular preventive dental services [64]. Some Puerto Rican parents consider eruption of teeth during the usually specified period reassuring of the child's normal growth and development [73]. Some Mexican-American migrant farm workers are said to have a fatalistic attitude toward health, including oral health [64,65], an idea possibly consistent with a claim that Hispanics/Latinos seek oral health care mainly in response to pain and concern over esthetic appearance [65]. Only one study, by Lopez and colleagues, [73] references Latino beliefs about the etiology of oral disease among infants as infection, bad breath or *sapo *(candidosis) [73]; Puerto Ricans believe that babies get infections in their mouth from milk. This study also suggests use of "pink honey" available over the counter to clean the baby's tongue. Cleaning the mouth with leaves of a plant, *la hoja del gandul*, was a folk remedy used in the past but now slowly disappearing [73].

## Discussion

While many epidemiological studies and clinical surveys suggest links between race/ethnicity and oral health status, actual cultural beliefs and values that influence decisions or practices regarding oral health are seldom reported. Much of the published literature on the oral health of ethnic minorities and other disadvantaged groups is epidemiological in nature. These accounts are important and make many key contributions to knowledge, often pointing to the importance of cultural issues as factors influencing the results being reported. As revealed by this assessment of the literature, few, if any, of these clinical, quantitative or epidemiological studies further specify the precise nature of the cultural issues alleged to be of relevance.

Relatively few studies have been published with a focus on culturally influenced beliefs and behaviors affecting oral health. Using the search strategy noted, we found almost nineteen hundred references for all four race/ethnic minority populations; however, only about 10% (246 references) were deemed relevant and were read in full. Of these, only a small proportion (60 in total; 23% of initially screened) met all the inclusion criteria for this study and were included in this review. Surprisingly, although Hispanics/Latinos and African-Americans are the largest racial/ethnic minority groups in the US there is still very limited literature on their health-related cultural beliefs and practices and the influence of these on oral health. The majority of included works referred to the Chinese population. As yet, almost no information links culture and oral health for the Filipino population.

This review is the first of which we are aware to summarize and evaluate the state of the literature in dental science sources related to cultural beliefs and practices and their influence on oral health. This literature search has identified several large gaps in knowledge about cultural influences on oral health and health care. It is important that these gaps be addressed if improvement in the oral health status of minority populations is to be achieved.

### Limitations

This review has some limitations. First, only four groups were included. This strategy excludes many other minority groups in the US and elsewhere. However, the four racial/ethnic population groups included here are populous with considerable heterogeneity within the groups by geographic location, and socio-economic status in particular. Three of these populations (Chinese, Filipino, and Hispanic/Latino) are growing rapidly not only in the US, but in other parts of the world which makes knowledge of their cultural beliefs increasingly important to oral health globally. Second, titles and abstracts when available, and not full texts, were used initially to identify works that discussed in some sustained way cultural values and their influence on oral health. A basic assumption was that information pertinent to a central discussion of cultural issues would be mentioned in the abstract. Information in the body of the text would not have been gleaned from articles with only incidental attention to this topic. Third, while some psychosocial electronic databases also abstract chapters in books associated with major publishing houses in their areas of interest, this is not the case for medical and dental literature. Hence, work published outside peer-reviewed mainstream medical or dental journals, in books or in sources not abstracted by PUBMED, was not reviewed. Hand searching was not undertaken, and misclassified articles were not retrieved. Thus some relevant articles may have been missed.

There is little reason, however, to think that this review about cultural influences on oral health would be vastly different if other nations or race/minority groups were chosen to anchor the investigation. The US is a large nation with a prolific and active research enterprise, yet even with that capacity surprisingly little systematic research attention has been paid to this topic. The four focal groups are demographically significant within the US and its most diverse state, California; several of these focal groups comprise significant minority populations elsewhere in the world, too. Yet, with the exception of (migrant) Chinese, the depth and extent of cultural knowledge relevant to oral health is very slim indeed. While some additional pertinent information can be found in sources outside dental science, in books and articles by social and behavioral scientists for example, frequently this work is not readily accessible, especially for clinicians working outside academic institutions. Even this information, however, is largely incomplete, scattered across multiple sources, and difficult to locate, especially if not catalogued in searchable databases or available in electronic format.

### Implications for future research

Qualitative research is often thought not to be suitable for evidence-based systematic reviews since it does not provide numerical values for point estimates. However, it does provide valuable information leading to meaningful conclusions wherever appropriate. An example quoted by Dixon-Woods and Fitzpatrick [76] demonstrates the value of qualitative research in evidence-based practice using a Cochrane review on how to improve communication skills with children and adolescents about their cancer. This analysis focused only on randomized controlled trials and before and after studies, and included only 6 of 1500 identified studies. Clearly, a more inclusive view of what constitutes evidence is necessary to answer complex questions or address important issues that cannot be easily quantified [76].

In the last two years, some studies in the dental literature have done exactly what we call for: namely, more thorough and rigorous studies of cultural influences on oral health. Some of these works report cultural influences on oral health practices and attitudes for Chinese, African-Americans, Hispanics and Filipinos [7,30-33,59-62,73,74]. These more recent articles have utilized qualitative study designs and explored underlying cultural reasons for seeking, or delaying treatment for children. While connections between these oral health-related findings and other aspects of culture are still nascent, these more recent works are valuable for their more detailed attention to these connections. Studies that report oral health status in conjunction with cultural beliefs are helpful for teasing out cultural influences from other potentially confounding factors, such as income, education level and other social determinants that are important predictors of dental health and disease.

A relatively new approach to public health research is community-based participatory research (CBPR) [77]. This intensely collaborative approach involves community members in all phases of the research process including problem-identification, development of culturally appropriate research methods, and engagement in data collection and interpretation as well as dissemination of findings. Federal funding for this type of research has been increasing recently [78]. CBPR should help identify, enhance our understanding of culturally important beliefs and practices and increase implementation of culturally appropriate, acceptable and sustainable interventions and services.

### Implications for designing future dental education and public health programs

The information gained from further research into cultural beliefs and its association with oral health and dental care seeking behavior and practices would be helpful in designing future preventive and treatment programs. Such programs with special considerations to ethnic beliefs could have a positive impact and increase utilization of preventive services. The US Surgeon General's Report [5] not only highlights disparities in oral health but also highlights the importance of social and environmental determinants of oral health and the need to adopt more holistic approaches to oral health promotion. A well-trained dentist should be not only need to be an expert in clinical skills but also able to elicit, recognize, accept and respect their patients' cultural beliefs. Professional dental care is increasingly conducted in multicultural environments. Since health care is a cultural construct arising from beliefs about the nature of disease and the human body, cultural issues are actually central in the delivery of health services treatment and preventive interventions. By understanding, valuing, and incorporating the cultural differences of America's diverse population and examining one's own health-related values and beliefs, health care organizations, practitioners, and others can support a health care system that responds appropriately to, and directly serves the unique needs of populations whose cultural beliefs, values and practices may be different from those of the surrounding dominant culture [79]. The diversity of ethnic groups in the US and elsewhere presents a challenge to both policy makers and oral health care providers who need to design and provide health care services to multiple populations. A deeper understanding of health behaviors as influenced by culture, health beliefs, acculturation, and attitudes is needed to formulate appropriate oral health promotion policies.

## Conclusion

We conclude that, overall, dental science's present knowledge about how culture influences a group's oral health exhibits several deficits. These comprise: first, a lack of conceptual clarity and careful specification of the population under examination; second, an absence of explicit theory and use of a limited set or inappropriate type of method of inquiry; and, third, an incomplete and sketchy coverage of pertinent topics and age groups. These deficits can and are being remedied. Recent published works have done exactly what we call for: namely, more thorough, rigorous studies of cultural influences on oral health.

Qualitative researchers engaged in ethnographic, interview and naturalistic observational studies are well trained and skilled in conducting studies about cultural beliefs and practices, and often have experience in working with community collaborative partners. They should be encouraged to collaborate also with clinicians and dental public health practitioners to further this important avenue to understanding patients, their treatment decisions and outcomes.

Studies designed to further understand the role of cultural beliefs, values and practices should be targeted very specifically. Investigation should not be limited to the study of oral health of a particular age group (e.g., the elderly, children) but should examine attitudes toward oral health for all, adults and children, and cover broadly ideas related to oral disease etiology, pain, orthodontia, motivation for seeking care, maintaining good oral health, and actual oral health preventive and remedial practices. Carefully crafted, thorough investigations will assist clinicians to elicit, recognize, accept and respect their patients' cultural beliefs, and to develop appropriate therapeutic strategies as well as oral health promotion policies.

## Abbreviations

PUBMED: US National Library of Medicine; TCM: Traditional Chinese Medicine; CBPR: Community Based Participatory Research.

## Pre-publication history

The pre-publication history for this paper can be accessed here:


